# Impact on Mental Health of Families during Covid-19: A Cross-Sectional Survey

**DOI:** 10.4314/ejhs.v31i6.7

**Published:** 2021-11

**Authors:** Sumaira Imran Farooqui, Amna Aamir Khan, Jaza Rizvi, Batool Hassan, Qurat-ul-Ain Adnan

**Affiliations:** 1 Ziauddin College of Rehabilitation Sciences, Ziauddin University

**Keywords:** Coronavirus, Anxiety, Depression, Family, Mental Health

## Abstract

**Background:**

The global pandemic of novel coronavirus outbreaks threatens the general public and health care workers' physical, social and mental well-being. Therefore, the current study is aimed to highlight the status of mental health of families suffering from the COVID-19 pandemic.

**Methods:**

An online cross-sectional survey was conducted on 560 families through Google Form distributed via email, Whatsapp groups, Facebook, and LinkedIn from November 2020 to January 2021 during the pandemic period in Karachi through the snowball sampling technique. The status of COVID-19 patients was assessed through demographics information and contextual factors questions whereas impact on mental health was assessed through Depression Anxiety Stress Scale (DASS-21).

**Results:**

A total number of 536 participated in the study. The prevalence of depression, anxiety and stress was found to be 38.99%, 26.67%, and 15.48% respectively included 31% of males and 69% where the highest number of respondents belonged to district Central (37.8%). Chi square estimation was found to be significant among both the gender and in between all the age group ranges from 20 to 60 years. Moreover, significant association among categorical division of demography and DASS-21 p<0.05 was observed.

**Conclusion:**

The results of our study indicated high rate of depression and anxiety in majority of families; in particular females. Consistent with these symptoms, significant association was found between gender and age from high to low socioeconomic status.

## Introduction

The global pandemic of novel coronavirus (COVID-19) outbreaks threatens the public and health care workers' physical, social and mental well-being (1). On 31^st^ December 2019, In Wuhan City, China appearance of pneumonia of unknown origin was the initiation of a breakout. This afterward came to be proclaimed as a pandemic by the World Health Organization (WHO). The term COVID-19 (Contraction for “Coronavirus Disease 2019”) was devised on 11^th^ February 2020 to explain presentation with severe acute respiratory illness (2). In an emergency meeting on 30^th^ January 2020, COVID-19 is declared as a global public health emergency by the WHO and Public Health Emergency of International Concern (PHEIC) (3). The novel coronavirus is a worldwide burden on the socioeconomic and health systems of the world. According to WHO data globally, 213 countries with 104,956439 cases are confirmed out of which 2,290,488 deaths have been reported till 4th February 2021. In Pakistan, WHO data confirmed 551,842 cases with 11.886 deaths reported till 6^th^ February 2021 (4). To control the disease transmission worldwide, safety measures have been implemented including countrywide lockdown, quarantine, social distancing as well as workplace, school, and university closures. These unprecedented measures have disturbed the emotional and psychological wellbeing but unfortunately, the actual number cannot be predicted (5,6). Multiple pieces of research at the national and international level have focused on major mental health challenges due to COVID-19 including anxiety, depression, panic attacks, stress, and fear of unemployment or contracting the virus, similar psychological distress were reported in the past during the influenza pandemic (7–10). Behavioral issues and mental health problem has been observed in this era due to stress, anxiety, and depression. The neuropsychiatric connections have been observed between the novel coronavirus and mental health illness (5). Studies conducted during the period of Middle East Respiratory Syndrome (MERS) outbreaks on psychological and stress factors showed that these outbreaks affect a person's sleep quality and markedly imposed psychological distress (11).

Researches addressing mental health during the tenure enforces the development of a multidisciplinary team comprising of a clinical psychologist, psychiatrists, psychiatric nurses, and other mental health care workers to facilitate the counseling session for patients, their families, and health care workers. Different modes of communication can be used to provide a safe environment in the era of isolation (12).

Multiple elements including quarantine, physical distancing, unemployment, caregiver stress, panic, stigmatization, and death in the families impose negativity among the primary health care providers and families, therefore coping mechanisms are required to improve the mental health. Special considerations shall be given to the elderly population and people with already diagnosed psychological issues in cases of prolonged isolation, quarantine, and distressing environment (10). The guidelines provided by WHO on mental health and psychosocial considerations in this pandemic also highlighted that maintaining the routine, diet, exercises and social interaction with other appropriate measures can improve the recovery road. Keeping healthy routines involving doing daily chores, painting and singing can help too during stressful situations (13).

The current study is aimed to explore the prevalence rate and factors associated with depression, anxiety, and stress among the family afflicted with their loved ones who have contracted COVID-19.

## Methods

This study was adhered to the STROBE guidelines and was approved by the departmental review committee of Ziauddin University with the reference number 0197120022DPT. An online cross-sectional survey was sent to 560 families and was ensured that the questionnaire is filled by participants aged 20 to 60 years between November 2020 to January 2021 during the pandemic period in Karachi through the snowball sampling technique. As per the recommendation of the Pakistani Government to maintain the social distancing, the questionnaire was used on the google form and was circulated on different platforms through email, Whatsapp groups, Facebook, and LinkedIn. The survey was conducted in only the English language and no translation or back translation was required.

Out of 560 families, only 540 families agreed to participate in the study, 4 families were excluded as they were from Lahore. An online written consent was taken from all the participants before answering the questions.

**Measures**

**Socio-demographic information**: The questions comprising of demographic information including age, gender, district of residence (Central, East, South, West), the highest level of education, occupation, type of family (Nuclear, Couple or Extended), and socioeconomic status (Upper, Middle, Low).

**Status of COVID-19 patients**: Questions related to the history of travel, number of contacted cases, number of confirmed cases, and their current recovery status were also asked.

**DASS-21**: The 21-item Depression Anxiety Stress Scale (DASS-21) was used to assess the mental health status of the people whose families suffered from COVID; either losing their family member or recovered. The scale is divided into three subscales: Depression, Anxiety, Stress and each has seven items; where every item subscale comprises of four responses reflecting the severity from did not apply to me (0) to apply to me most of the time (3). The score ranges from 0 to 63, where the higher score indicates more symptoms with cut-offs of depression≥10, anxiety≥8, and stress≥16. DASS-21 has previously been used among research related to COVID-19 to assess mental health (14–16). The scale is a valid screening tool with excellent reliability for the subscales of depression, anxiety, and stress respectively (Cronbach's alpha=0.81, 0.89, and 0.78) (17).

## Results

A cross-sectional study was conducted on 536 participants with an overall response rate of 95.71% whose family members suffered from COVID-19 during the pandemic in which their levels of depression, anxiety, and stress were estimated. The prevalence of depression, anxiety, and stress was found to be 38.99%, 26.67%, and 15.48% respectively among the population that included 31% of males and 69% of female participants. Where the highest number of respondents belonged to district Central (37.8%) followed by East (24.4%) while the lowest participants were from district West (5.9%). In particular, 49.6% of participants confirmed their contact with the cases however 26.7% were not aware of their contact with confirmed cases. It was further analyzed that out of a total number of participants 9.7%, 4.29%, and 9.7% were found to be extremely depressed, anxious, and stressed respectively during the pandemic as depicted in Table-1.

**Table 1:** Frequency and percentage of participants in different categories of DASS-21

Variables	Normal	Mild	Moderate	Severe	Very severe
**Depression**	32 (5.97%)	55 (10.26%)	29 (5.41%)	42 (7.83%)	52 (9.7%)
**Anxiety**	40 (7.46%)	18 (3.35%)	25 (4.46%)	37 (6.90%)	23 (4.29%)
**Stress**	29 (5.41%)	27 (5.03%)	13 (2.42%)	9 (1.67%)	5 (0.93%)

*DASS-21-Depression Anxiety Stress Scale, *Cut-off scores-Depression≥10, Anxiety≥8, Stress≥16

Chi-square estimation of the data provided evidence that the level of depression, anxiety, and stress were found to be significant among both the gender and in between all the age group ranges from 20 to 60 years (mean age 33.45±6.3). The analytical analysis further confirmed the findings that the level of depression, anxiety, and stress were found to be non-significant independent irrespective of age and gender as the value of a coefficient of correlation R^2^ were found to be non-significant p>0.05 and the regression curve was also found to be independent between age, and the level of depression, anxiety and stress confirming the fact that COVID-19 pandemic affected population of all age group equally as shown in Table-2.

**Table 2:** Demographical association of age and gender with DASS-21

Variables	Age	Gender	Pearson co- efficient of correlation	p-value	Regression	p-value	Chi square estimation	p-value
**Depression**	20–60	Both	-0.09	0.25	0.098	0.12	29.65	<0.05
**Anxiety**			-0.04	0.64	0.04	0.64	1.22	>0.05
**Stress**			0.063	0.46	0.063	0.23	53.98	<0.05

Cross-tab analysis between the type of family and socioeconomic status with the levels of depression, anxiety, and stress as depicted in Table-3 revealed a significant association among categorical division of demography (Type of family and socioeconomic status) and DASS-21 p<0.05. Moreover, it was also observed during the analytical evaluation of the data that the middle socioeconomic strata of the society were scored greater in DASS-21 as compared to low and high socioeconomic classes.

**Table 3:** Cross tab analysis of type of family and socioeconomic status with DASS-21

Variables	Chi square	Type of family (p-value)			Socioeconomic status			

		Nuclear	Couple	Extended	Upper	Middle	Low	p-value
**Depression**	10.66		<0.05		11.19%	16.04%	11.75%	
**Anxiety**	3.22		>0.05		9.51%	12.68%	4.47%	<0.05
**Stress**	15.66		<0.05		6.15%	5.22%	4.10%	

## Discussion

The COVID-19 pandemic is the largest among realms of diseases in modern history affecting societies and economies in unprecedented ways. This survey was conducted on 536 families across different district of Karachi to evaluate the impact of the pandemic measures on mental health. It was revealed that symptoms of depression and anxiety was high in most of the families. Consistent with these symptoms, significant association was found between gender and age ranges from 20–60 years from high to low socioeconomic status. However, not everyone was suffering amid crisis, as nearly 20% of participants who reported consistent level of mental health with flourishing. Moreover, current outcomes didn't suggest dire consequences as observed in previous samples of families with selective or quarantined individuals due to possible ways of coping with difficult situations (18). Previous studies on deadliest pandemic like Severe Acute Respiratory Syndrome (SARS), MERS and Ebola had shown severe emotional distress among individuals in association to disease outbreak (19). Unfortunately, there is scarce evidence on past outbreaks to measure its impact on mental health; nevertheless, certain parallels can be drawn. The situation of COVID-19 is comparable with SARS and MERS as number of studies has found that moderate level of social, emotional and psychological wellbeing is associated with increased subsequent lingering health problems and healthcare expenditure (18, 20).

Our study observed that different age groups depicts different behavior due to short and long term effects, as individuals may experience stigmatization and develop a mix of emotions who recently released from quarantined (21). Similarly, people who have recovered were noticed to have exercise social distancing from their immediate family members, friends and relatives to ensure their safety due to unprecedented viral nature of disease (22). In addition to it, separation from family/friends, being aware of their illness or fear of losing loved ones may leads to undesirable adverse effects on psychological health that may precipitate the development of tremendous level of anxiety, stress, depression or other mental illnesses (23).

Several studies reported that COVID-19 may have an adverse impact on children's mental health in particular due to fear of virus affecting their families and surroundings also cause uncertainty in their academic performance and ambitions that pose a significant threat to their mental being that may impose greater risk of anxiety, depression and post-traumatic stress disorder (19,24,26). Despite of the fact, elderly people aged 60 or above were found to be more prone to the COVID-19 due to clinical and social reasons (27). As elder people are dependent on their families for their daily needs therefore physical distancing or isolation may induce a traumatic situation for them (28).

Studies indicated that survivors from previous pandemics showed worsen depression that corresponds to increased suicide ideation, neuropsychiatric illnesses and low quality of life (29–31). Therefore, recent and ongoing surveys are worthwhile to considerate immediate or short-term impact of direct factors at a certain point of time during pandemic that could pose emotional disturbance (32). These may include several weeks of home stay that has forced many individuals to work-from-home (33). Besides, many families have also lost their jobs due to which some have struggled to feed their children, also not all families with resources were able to adequate supplies (34). Thus, it is assumed that the reach of pandemic to families is unequal on the basis of their situation. Furthermore, in consideration to existing vulnerabilities, high risk factors and erratic stressors to ongoing pandemic may be changeable in corresponding to apparent outcomes (35).

As physical and psychological health care interdependent therefore it is crucial to mitigate distress during early stages of disease-related crisis to assist affected individuals in coping with long-term impact of stress on mental health (36). The well-being of families may directly impact their mental health therefore it is vital to encourage the adoption and maintenance of health-related behaviors among them (37). The WHO has urged people to follow better hygiene practices, social distancing guidelines and use of appropriate protective gear especially from the showcasing individual (37). Moreover, regular physical activity, healthy eating and avoiding alcohol or drugs are also crucial to minimize the anxiety and fear of the pandemic (37). Furthermore, healthcare organizations can also implement psychological guidelines to assist families with mental distress within communities to provide psychosocial support to any survivors of pandemic (37).

Amid time of paramount stress that has a direct or indirect impact on mental health of people across the world, the role of mental healthcare workers is crucial to challenging situations with professional responsibilities (37). Also, it will be crucial to prevent post-pandemic mental health ailments in underprivileged unit of society. Thus, the focal point of the healthcare system should be disease prevention and health promotion corresponding to health needs of the population at large, considerate to regional contextual parameters for the prompt management of mental health morbidity (32).

This survey provides valuable insights to document mental health outcomes in Karachiites during COVID-19 pandemic. Moreover, it informs the contextual factors within families that affect the mental health outcomes. Besides, several limitations were encountered due to certain factors that are outcomes are comprehended on the basis of cross-sectional analysis and associations yet causation due to early and delayed impact of pandemic was not inferred. Moreover, survey results were obtained via self-reported answers that may be subjected to response bias yet the sample wasn't large enough to be the representative of population. Further, the population was under sampled due to time constrained data collection and selection criteria. Consecutively, future studies are encouraged to investigate resilience aspects as a transdiagnostic factor to evaluate the impact of mental health outcomes. The overall indulgent about COVID-19 has been expanded across the globe yet its impact on mental health on families with pandemic survivors is challenging to estimate due to uncertain situations that may pose greater risks to the psychological well-being. The results of our study indicated high rate of depression and anxiety in majority of families, in particular females. Consistent with these symptoms, significant association was found between gender and age from high to low socioeconomic status. Future studies should be conducted to investigate the risk factors to mental wellbeing to avoid detrimental consequences of this pandemic on the psychology of existing and current race.
